# Using light-dependent scleractinia to define the upper boundary of mesophotic coral ecosystems on the reefs of Utila, Honduras

**DOI:** 10.1371/journal.pone.0183075

**Published:** 2017-08-15

**Authors:** Jack H. Laverick, Dominic A. Andradi-Brown, Alex D. Rogers

**Affiliations:** 1 Department of Zoology, University of Oxford, Oxford, United Kingdom; 2 Operation Wallacea, Old Bolingbroke, Spilsby, Lincolnshire, United Kingdom; Leibniz Centre for Tropical Marine Research, GERMANY

## Abstract

Shallow water zooxanthellate coral reefs grade into ecologically distinct mesophotic coral ecosystems (MCEs) deeper in the euphotic zone. MCEs are widely considered to start at an absolute depth limit of 30m deep, possibly failing to recognise that these are distinct ecological communities that may shift shallower or deeper depending on local environmental conditions. This study aimed to explore whether MCEs represent distinct biological communities, the upper boundary of which can be defined and whether the depth at which they occur may vary above or below 30m. Mixed-gas diving and closed-circuit rebreathers were used to quantitatively survey benthic communities across shallow to mesophotic reef gradients around the island of Utila, Honduras. Depths of up to 85m were sampled, covering the vertical range of the zooxanthellate corals around Utila. We investigate vertical reef zonation using a variety of ecological metrics to identify community shifts with depth, and the appropriateness of different metrics to define the upper MCE boundary. Patterns observed in scleractinian community composition varied between ordination analyses and approaches utilising biodiversity indices. Indices and richness approaches revealed vertical community transition was a gradation. Ordination approaches suggest the possibility of recognising two scleractinian assemblages. We could detect a mesophotic and shallow community while illustrating that belief in a static depth limit is biologically unjustified. The switch between these two communities occurred across bathymetric gradients as small as 10m and as large as 50m in depth. The difference between communities appears to be a loss of shallow specialists and increase in depth-generalist taxa. Therefore, it may be possible to define MCEs by a loss of shallow specialist species. To support a biological definition of mesophotic reefs, we advocate this analytical framework should be applied around the Caribbean and extended into other ocean basins where MCEs are present.

## Introduction

Mesophotic coral ecosystems (MCEs) represent a distinct environment from shallow-water tropical coral reefs; primarily in terms of lower light levels and increased depth. In 2008 MCEs were defined as starting at 30-40m and extending to the depth of occurrence of the last zooxanthellate scleractinian [1], this definition has been widely adopted [2,3]. However, the use of an absolute 30m depth limit by many MCE researchers [4–12] potentially misses site-specific patterns in environmental variables; which may alter the character of a coral reef and so change the depths at which MCEs occur. There has been limited effort to identify whether a reef community can be identified as an MCE in the absence of depth information [13]. This is despite the recognition that the lower extents of MCEs vary by region, based on environmental conditions [14].

Previous work has found evidence of changes in photosynthetic coral reef community composition at a variety of depths [12,15–17], as well as observations of unusual or unexpected reef communities at particularly shallow or deep locations [18,19]. This has led to calls to rethink the static definition of MCEs [3,20]. In some cases, the differences in detected transitions in ecological communities across the depth gradient may result from different study taxa [13], in others because of factors specific to the study sites such as the availability of hard substrata and topography [15]. A third source of variation in transitions, previously little considered in MCE literature, may lie in differences in analyses employed to detect changes in reef communities.

The use of a static upper depth limit for MCEs by some appears to be at odds with these observations, and the methods for defining ecosystems in other areas of ecology. Different forest ecosystems may be defined by predominant tree species while deserts are defined in terms of rainfall [21]. Within marine ecology, community structure in the intertidal zone is driven by tidal water movements and exposure to wave action [22] which varies between sites. Different shallow reef zones such as the flat and crest have been recognised to differ in terms of ecology while often occurring at similar water depths [23]. Such definitions recognise variability in prevailing conditions at a given location, often using community data as a surrogate for integrated environmental parameters. This sort of approach allows for the design of natural experiments [24] where we can compare the effects of varying conditions on a particular biological assemblage.

More specific to MCEs, the Deep Reef Refugia Hypothesis (DRRH) suggests MCEs may provide a source of recruits to repopulate shallows reefs in the event of impacts on the latter [10,25]. For the DRRH to be correct, MCEs must have a degree of community overlap with the shallows. If conditions vary between sites, and determine the rate of species turnover with depth, this will affect the size of any species refuges on deeper reefs. Faster rates of transition with depth from a shallow community through to a mesophotic community would reduce the vertical space occupied by shallow reef species. This reduction in depth range would reduce the likelihood that shallow taxa exist at depths suitably large to afford protection from near surface environmental or anthropogenic stressors. In addition, MCEs face many threats in their own right, and clearer identification of what composes a MCE regardless of depth is crucial to inform conservation decision-making [26].

To try and address these problems, it is necessary to consider a method of defining MCEs which accommodates the variable nature of biology. There are, however, several different approaches which may be employed (Table 1). Here, firstly, we report on a study of zooxanthellate scleractinian coral communities from Utila, Honduras ranging from shallow reefs down to, what we consider to be, the maximum depth for this grouping at this location. Secondly we use this dataset as a case study in detecting signals in community composition for defining the vertical zonation of the reefs, between different statistical methods. Finally, this study suggests a method of identifying MCEs and the zonation within them that may be broadly applicable regardless of geographic context.

**Table 1:** Different potential bases for a definition of MCEs.

Information Base	Benefits	Weaknesses
Absolute Depth limits [4]	Easily implemented and consistent.	Fails to capture between site variability in environmental conditions. Remains biologically unjustified.
Light levels [27]	Accurately captures the proposed driving force of zooxanthellate coral reef structure. Mesophotic contains ‘photic’, which means ‘relating to light’. It could be argued light levels should be integrated into any definition. Other ecological zones in marine science are defined based on light attenuation (eg. euphotic zone).	Reef structure may lag behind changes in environmental conditions by years. Light levels and penetration profiles vary daily. An average signal requires a long observation period to be representative.
Indicator species [28]	Integrates a number of environmental signals over long time scales. Does not require extensive taxonomic knowledge.	Indicator taxa may not be shared between sites. What does a MCE indicator taxon truly reflect?
Biodiversity measures (e.g. species richness or evenness)	Integrates a number of environmental signals over long time scales. Metrics are taxon identity-independent so may be applied globally	Misses taxon turnover.
Community composition [29]	Integrates a number of environmental signals over long time scales. Incorporates species turnover. Allows the detection of different types of MCE.	Data intensive. Requires detailed taxonomic knowledge. Requires standardisation of analyses and agreement on prioritisation of rare or common taxa.
Growth forms [30]	Integrates a number of environmental signals over long time scales. Easily recognized by the non-specialists. May be applicable globally. Incorporates turnover of forms.	Different regions have different pools of growth forms. Certain taxa are phenotypically plastic and vary in growth form.

This list is not exhaustive and there are multiple ways to analyse data sources which may improve their utility. It is valid to argue for using a combination of data types.

## Methods

### Study site

**Fig 1:**
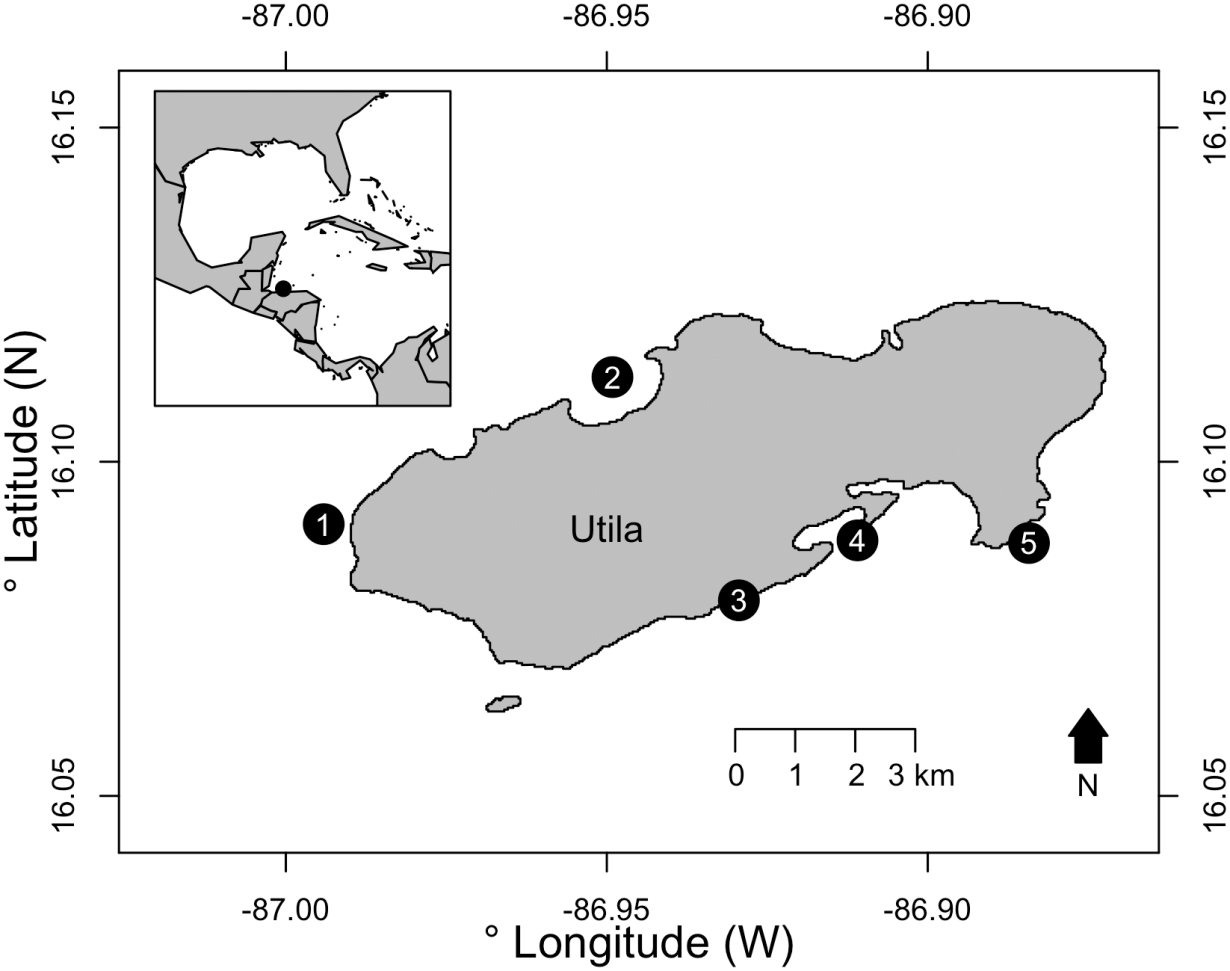
Dive site locations around the island of Utila, Honduras, Caribbean. Sites are listed with GPS co-ordinates and abbreviations starting left in WGS84 format: (1) Raggedy Cay (RC: N 16.09065964, W -86.9941015), (2) The Maze (TMA: N 16.11266214, W -86.94911793), (3) Little Bight (LB: N 16.07926302, W -86.92942222), (4) Coral View (CV: N 16.08823274,W -86.91094506), (5) Rocky Point (RP: N 16.08784039, W -86.88423403). The base map was sourced from GADM database of Global Administrative Areas under a CC BY licence with permission.

Utila is one of the Honduran Bay Islands, on the southern boundary of the Mesoamerican Barrier Reef in the Caribbean. The Island’s approximate population of 8,000 people is centred around Sandy Bay on the south shore [31]. The shallow south shore reefs exhibit a spur-and-groove system, which slopes to a sandy bottom at approximately 30-45m depth where the reefs form a patch reef system. The slope continues to descend gradually to approximately 70m depth towards mainland Honduras. The north shore reefs are primarily steep walls descending deeper than 100m, with an extensive shallow back-reef environment. Five sampling sites were chosen for their spread around the island (Fig 1).

### Data collection

50m video transects were collected by divers using mixed-gas closed-circuit rebreathers (Hollis Prism 2, Hollis, San Leandro, California, USA). Research permits were granted by the Instituto de Conservación Forestal, Honduras (Permit number: ICF-261-16), through Operation Wallacea. A Veho K2 action camera (Veho, Southampton, UK) in a 100m depth-rated housing was aimed down at the sea floor 20 cm from the bottom with a dive torch for illumination. Sample depths were 5, 15, 25, 40, 55, 70 and 85m, with four replicate transects collected at each depth following the respective depth contour at each site. Two transects were collected reef-on-left, and two reef-on-right starting 10m from the GPS location (Fig 1) with 10m between adjacent transects. The Utila south shore sites, Little Bight (LB) and Coral View (CV), reach a maximum depth of approximately 45m. The south-easterly site, Rocky Point (RP), levelled at approximately 63 m depth. The north shore sites, Raggedy Cay (RC) and The Maze (TMA), are located on a reef wall punctuated with shelves extending past 100m depth. These sites were sampled to 55m and 85m depth respectively as exploratory dives found no scleractinian cover at greater depths.

### Data analysis

Videos were analysed following scleractinian visual identification resources [32–38]. The bottom cover under the transect tape was identified every 25cm. Scleractinian corals, as well as *Millepora*, hereafter included with Scleractinia for brevity, observed 10cm either side of the tape were recorded in preference to other benthic cover types. This was justified as the primary concern was scleractinian community composition, and this approach maximised collected information. However, this method does produce an over-estimate in scleractinian percentage cover, especially when in low abundance. Identification was to species level when possible.

Percentage cover of broad benthic categories; Scleractinia, soft coral, macroalgae, coralline crustose algae, sponge, and sand, were calculated to detect the changing benthic composition with depth at all sites. The identified scleractinian data formed a species by transect matrix of counts. Transects recording no Scleractinia were removed to allow the generation of an ordination. Counts were used instead of percentages to prevent elevating the importance of rare species in the analysis. A lone observation of a species on a transect will appear more distinct to an ordination if recorded as comprising 100% of the community as opposed to an abundance count of 1.

Non-metric Multi-Dimensional Scaling (NMDS) [39] was used to generate an ordination with the packages Vegan and MASS [40,41] in the programming language R [42]. All results are reported as per the software output. All transects were plotted based on Bray-Curtis dissimilarities generated with scleractinian species data to visualise patterns between sites and depths.

To detect potential boundaries between shallow and mesophotic scleractinian assemblages, multiple Principal Coordinate Analyses (PCoA) were conducted using different levels of taxonomic resolution: species, genus and family. While NMDS provides a robust start point for visualisation, PCoA was used to allow further analyses conducted in Euclidean space. The data was Hellinger transformed to allow for the use of a method based in Euclidean space, standardising observations of each species by its total abundance across all transects [43]. Conceptually, species are plotted as points in as many dimensions as there were transects. Closely plotted species have more similar patterns of occurrence. The Euclidean distances between points can be considered as measures of dissimilarity. Points were assigned to an optimal number of groups, indicating distinct species assemblages. Communities observed on transects were statistically compared to these assemblages to see whether depth and site segregate communities spatially. The depth profiles of taxa were plotted against groups to illustrate the allocation of generalist and specialist taxa with respect to depth.

K-means clustering [44] was used to generate clusters to overlay on the PCoA. The optimal number of clusters was determined by trialling multiple potential clustering solutions and selecting the number of clusters which maximised the Calinski criterion [45]. Clusters returned were tested statistically using a multi-response permutation procedure with 999 iterations [40,46]. Dufrene-Legendre indicator species analyses [47] were conducted using the R package labdsv [48]. The analysis was performed on the transect data to determine which depths at which sites revealed patterns of co-occurrence in these clusters. Maximum indicator values occur when all Scleractinia observed on a transect have been previously assigned to a single cluster, which we interpret as an assemblage. The depth profiles of taxa were then compared to cluster occupancy to determine whether the structuring of reef communities is driven by depth-generalists or depth-specialists.

To compare other methods of detecting reef zonation, the family Agariciidae was analysed in isolation as a potential indicator taxon. Species and genera richness were recorded across all sites and Shannon’s diversity indices were calculated for depths, trend lines were fitted. The choice was limited to considering the use of a linear, logarithmic, exponential or second order polynomial fit to prevent over-fitting. The equation which maximised *R*^2^ was selected. This equation was differentiated and the result plotted to reveal rates of change with increasing depth. Raw data for all analyses can be found in supplementary data (S1 and S2 CSV).

## Results

**Fig 2:**
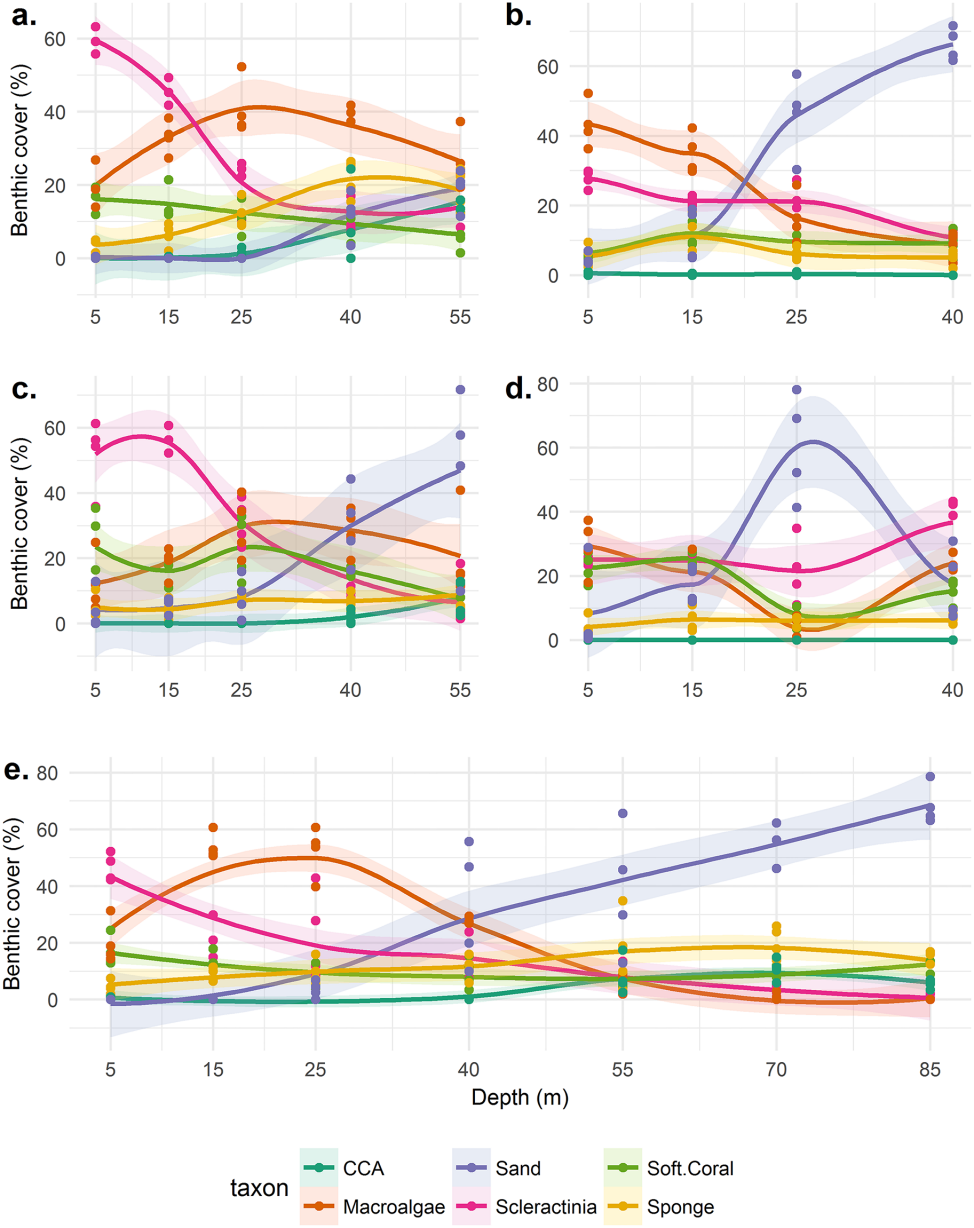
Benthic percentage cover by category at each sampling site. Each site was sampled with four replicate transects at each depth except 5m at RC which had three. Trends are plotted with a loess line and their 95% confidence intervals. CCA = Coralline Crustose Algae. Panels represent different sites: (a) RC, (b) CV, (c) RP, (d) LB, (e) TMA.

Patterns of benthic cover with depth appear dependent on site (Fig 2). At Coral View increasing depth led to a decrease in scleractinian cover and macro-algae. The same applied at The Maze with the addition of a distinct lag between the reduction of algae behind the reduction in scleractinians and an increase in octocoral cover with increasing depth. At Little Bight live benthic cover remained constant in all categories except for a belt of increased sand cover at 25m which breaks the reef structure. Raggedy Cay and Rocky Point had persistently high macro-algal cover while octocorals and scleractinians both decreased with depth. All sites showed an increase in sand with increasing depth indicating a reef structure which becomes increasingly fragmented.

**Fig 3:**
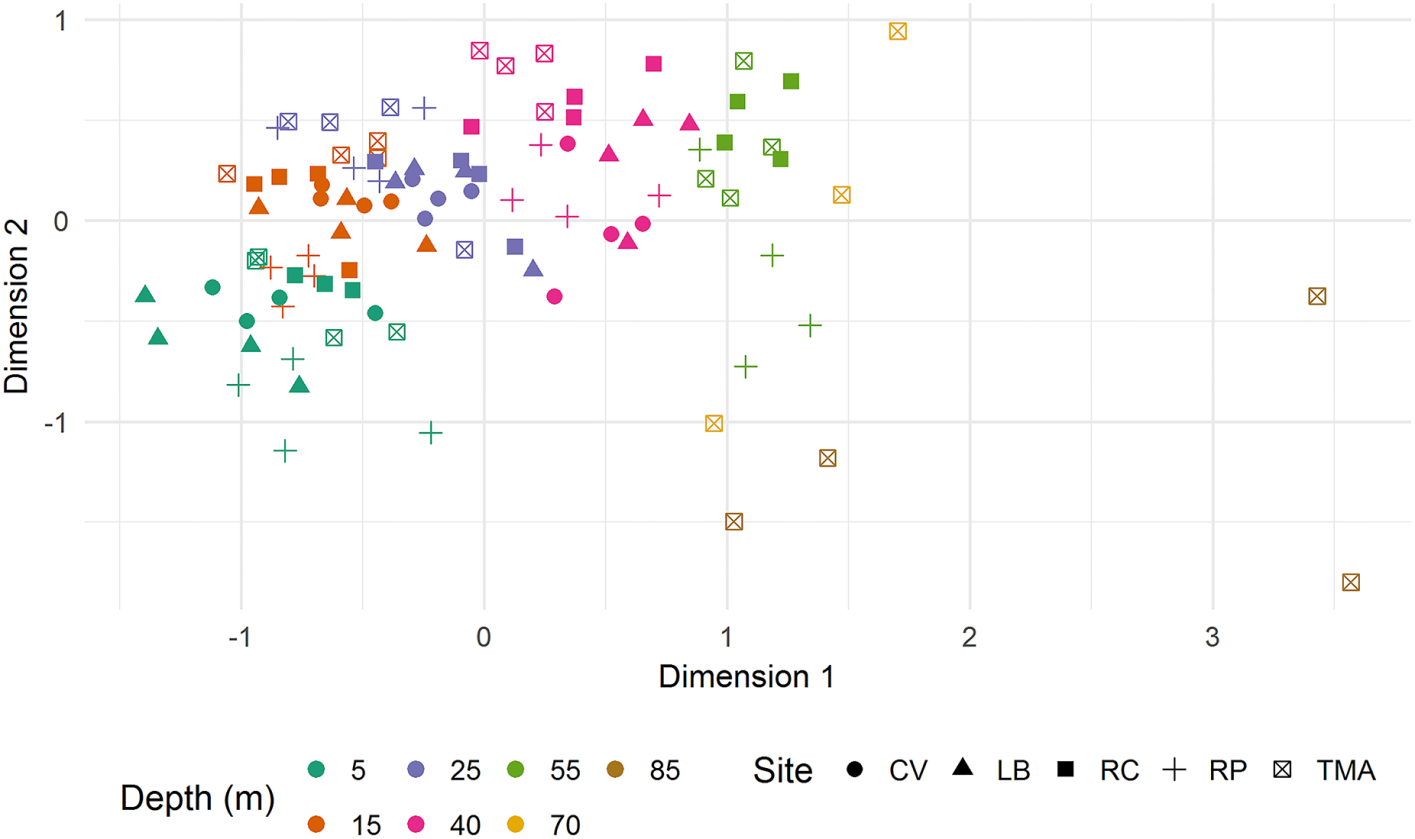
Transects discriminated by observed scleractinia communities. NMDS plot of transects based on Bray-Curtis dissimilarities from Scleractinia species counts. Symbols denote site and colours depths.

In all, 41 scleractinian species were documented (S1 Table). A non-metric fit to a Shepard plot (S1 Fig) returned an *R*^2^ = 0.976, and the stress of the NMDS plot = 0.155 2 s.f. suggests an informative amount of variation is presented in Fig 3. Variation along the first NMDS axis appears to correlate with increasing depth, with depth bands appearing clustered (Fig 3). The increased spread of points at the deepest sampling points is possibly an artefact of low counts. There are hints of varying rates of community change with depth between sites. For example, the 15m Rocky Point transects group with 5m transects for other sites, and three of the 25m Rocky Point transects appear to sit within a cluster of 15m transects from other sites. There appears to be no evidence for a clear two clusters marking shallow and mesophotic species.

**Fig 4:**
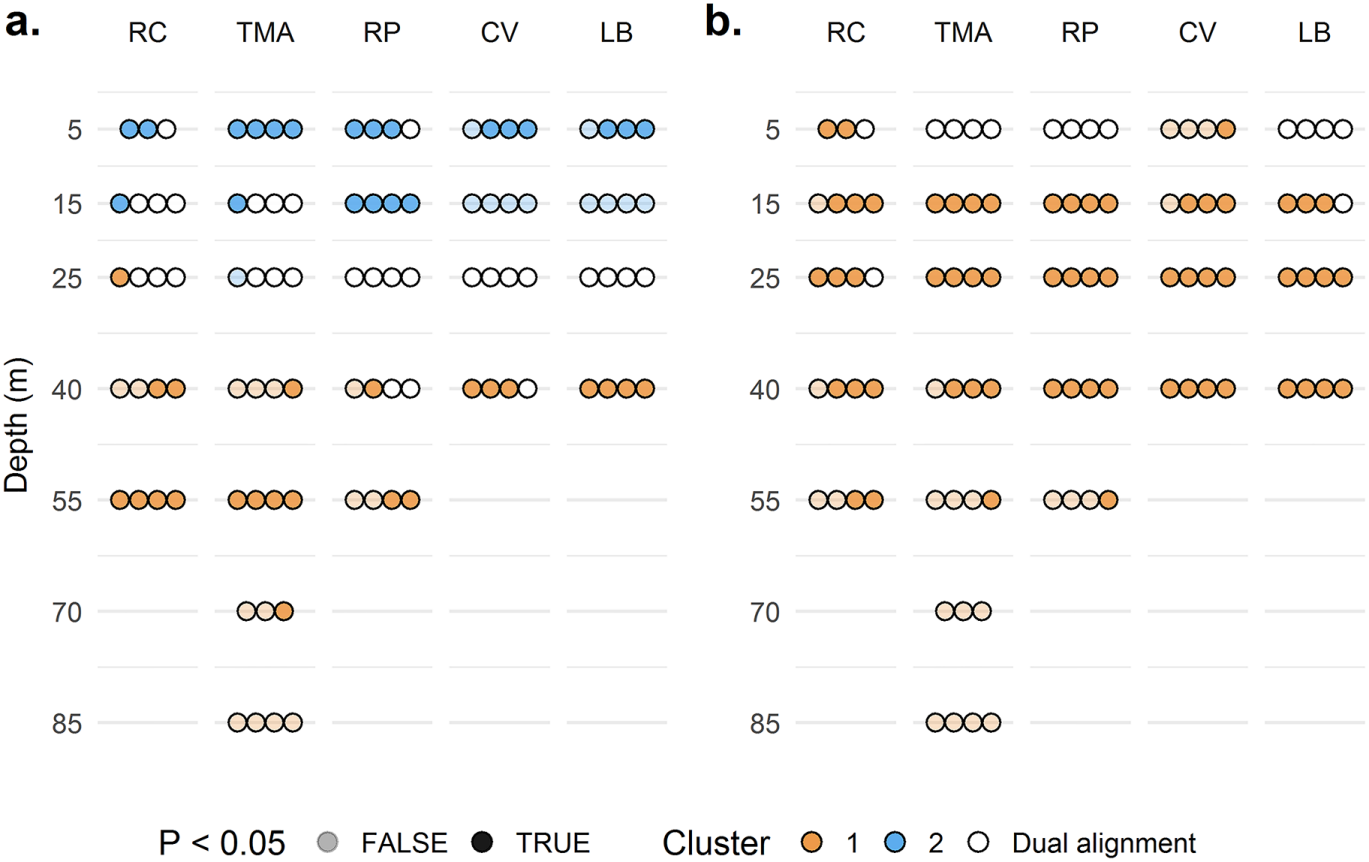
A map of assemblages at transect locations. Circles have been coloured based on cluster assignment of a transect at the indicated site and depth, with four transects (circles) per depth at each site. The number of the cluster relates to the analyses explicitly presented in S2 Table. Dual alignment was assigned to any transect for which R provided an indicator value for more than one cluster. There are 4 transects visualised per depth at a site. One 70m transect at TMA was removed as it captured no Scleractinia. The video file for one 5m transect at RC was corrupted. (a) Analysis based on species level ID. (b) Analysis based on genus level ID.

To further explore the structure of scleractinian communities PCoAs were generated. For species- and genera-level analyses the Calinski criterion (CC) increased monotonically, with the highest value supporting two clusters of taxa with values of 6.6 and 5.0, respectively (Panels a-b in S2 Fig). A multi-response permutation procedure testing for statistical significance of clusters returned *P* = 0.006 and 0.042 for two clusters at species and genus level, respectively. Family-level analyses revealed an erratic relationship between cluster number and CC presenting two peaks (Panel c in S2 Fig). Seven clusters maximised the Calinski criterion (CC = 5.4, *P* = 0.881). Dufrene-Legendre indicator species analyses determined which transects were representative of defined assemblages of taxa. Transects significantly associated with clusters are displayed in S2 Table along with *P*-values. Fig 4 presents the data visually, picking out the locations where the species assemblages occur.

Fig 4a indicates that the assemblages detected segregate by depth, when data are analysed at the species level. There are significant shallow and deep assemblages. In the middle exists a transitional zone (white) equally ascribed to either assemblage. Note this is not a statistically supported group in its own right. In hashed colours are transects containing a single assemblage when *P* >0.05. More specifically there appears to be a differing rate of transition with increasing depth between sites. Our results suggest the south shore sites Coral View and Little Bight show distinct deep assemblages at 40m. It is not until 55m the north shore sites Raggedy Cay and The Maze show similar significant deep assemblages. In contrast, Rocky Point shows a statistically significant shallow grouping at 15m, deeper than at other sites, and doesn’t consistently form patches of reef with the deep assemblage. The lack of significance of the signal at The Maze at 70 m and 85 m may be an artefact of low cover reducing power of the analysis.

The genus-level analysis was able to detect two statistically significant clusters, yet when mapped onto locations (Fig 4b) the only signal visible is a single assemblage with a broad depth range. This assemblage had a large spread across all sites, losing statistical significance past 55m depth. 5m appeared to represent a transitional zone. The family-level analysis maps were uninformative, possibly resulting from the large number of clusters lacking statistical significance.

**Fig 5:**
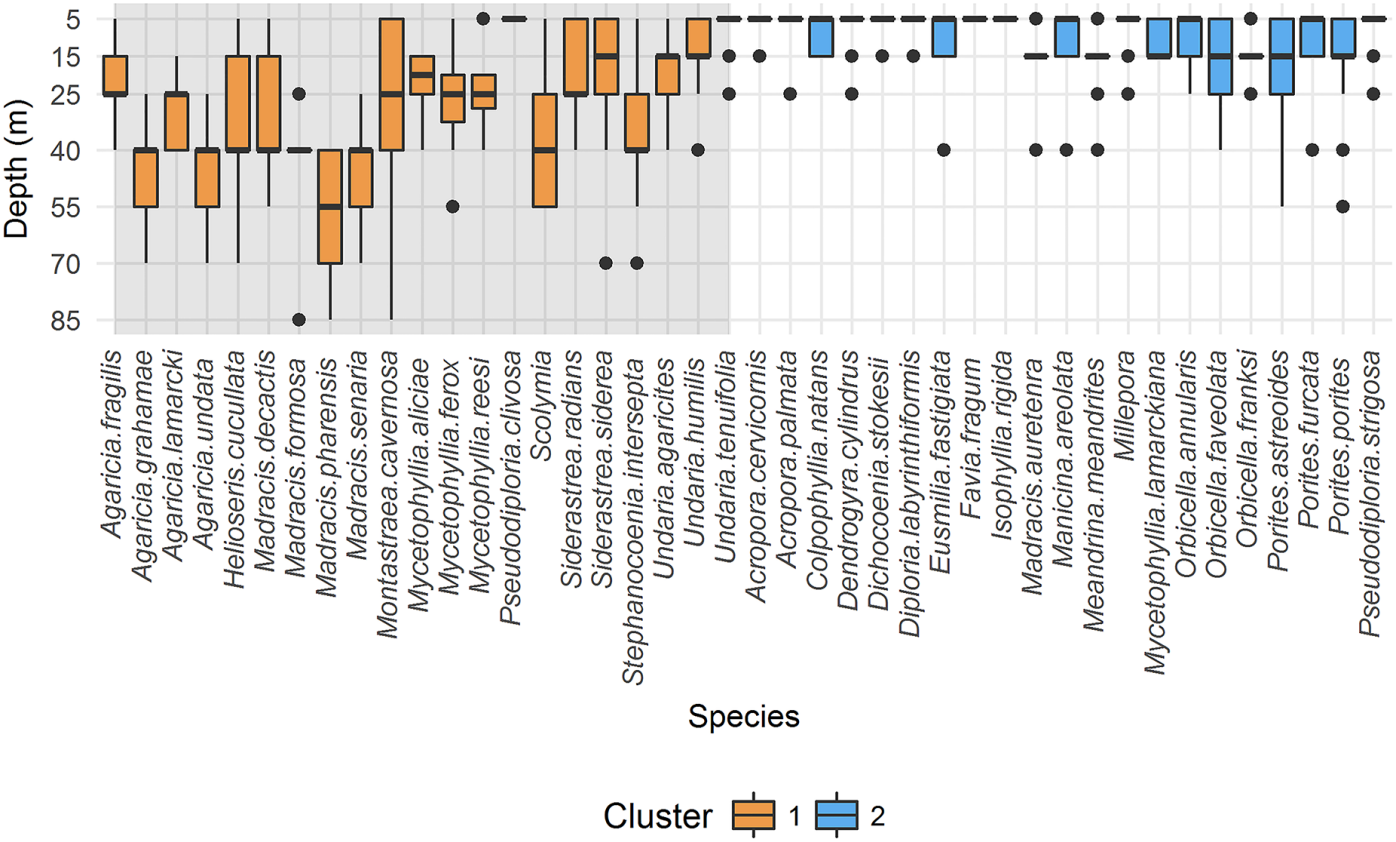
Dataset average depth ranges of scleractinia species. Box plots of species depth ranges generated from pooled data of all sites. Box plots are coloured internally to reveal the assemblage they belong to. All box plots on the darker background belong to Cluster 1, the lighter background denotes cluster 2. Lines extend 1.5x the interquartile range or to the last observation. Points are outliers beyond this limit.

Fig 5 reveals the two different species assemblages determined by the above analysis across all sites. It appears the shallow assemblage is characterised by shallow-water specialists such as *Acropora cervicornis* and members of the genus *Orbicella*. The deep-water assemblage consists primarily of depth-generalist species. *Madracis pharensis* is the only species in the deep cluster not detected shallower than 40m. *Agaricia grahamae*, *Agaricia undata*, *Madracis formosa* and *Madracis senaria* are the next deepest, with ranges starting at 25m. The transition zone depicted in Fig 4a was characterised by a loss of shallow-water specialists and increasing dominance in depth-generalists. This suggestion is supported by Fig 4b only mapping a single assemblage with a broad depth range, only giving way to a transitional signal at 5m. This is what would be expected if the statistically significant deep cluster reflected all depth generalists, a signal which becomes diluted at 5m by the appearance of shallow specialists. A similar plot of depth distributions for genus-level analysis by cluster can be found in the supplementary material (S3 Fig). The general pattern described with a shallow-specialist and depth-generalist community appears to hold true for genus level distributions (S3 Fig). Exceptions are the genus *Agaricia* aligning with the shallow assemblage and *Undaria* and *Colpophyllia* with the depth-generalist assemblage. The lack of statistical significance and a large number of mono-family clusters removes the ability to discuss patterns in the family-level analysis.

Following these results, *a posteriori* analysis of agariciid corals as a putative indicator taxon was performed. The Agariciidae exhibit a large depth range as a family and were relatively common across all depths. The methodology followed was the same as applied to the species-genus-family analyses. Five clusters appeared optimal, CC = 3.1, *P* = 0.566, but again there were two peaks. As *Helioseris cucullata* and *Agaricia fragilis* formed mono-specific clusters these were removed and the analysis re-run. This was in case a large number of clusters hid a signal, as may have been the case with the family- level analysis. Three clusters were found with CC = 4 at a single peak and *P* = 0.067. Defrene-Legendre indicator species analysis failed to return any transect aligning with a single cluster of statistical significance. The three clusters were *Undaria tenuifolia* and *Undaria humilis*, *Agaricia lamarcki* and *Undaria agaricites*, *Agaricia grahamae* and *Agaricia undata*.

To explore whether the patterns in Fig 4 reflect a loss of shallow specialists and the maintenance of a depth generalist community, rather than the gain of depth specialists, a non-parametric Mann-Whitney U test was performed. The depth ranges of species in both clusters were compared. Outliers were defined as the few observations falling further than 1.5 times the interquartile range from the median depth value of the species; these were excluded from the analysis. The results (*W* = 68, *P* < 0.0001, with 21 species in each group) show there is a significant difference. The mean depth range of the depth generalist cluster was 35.2m ±20.4m while for shallow specialists, it was 8.3m ± 13.4m.

**Fig 6:**
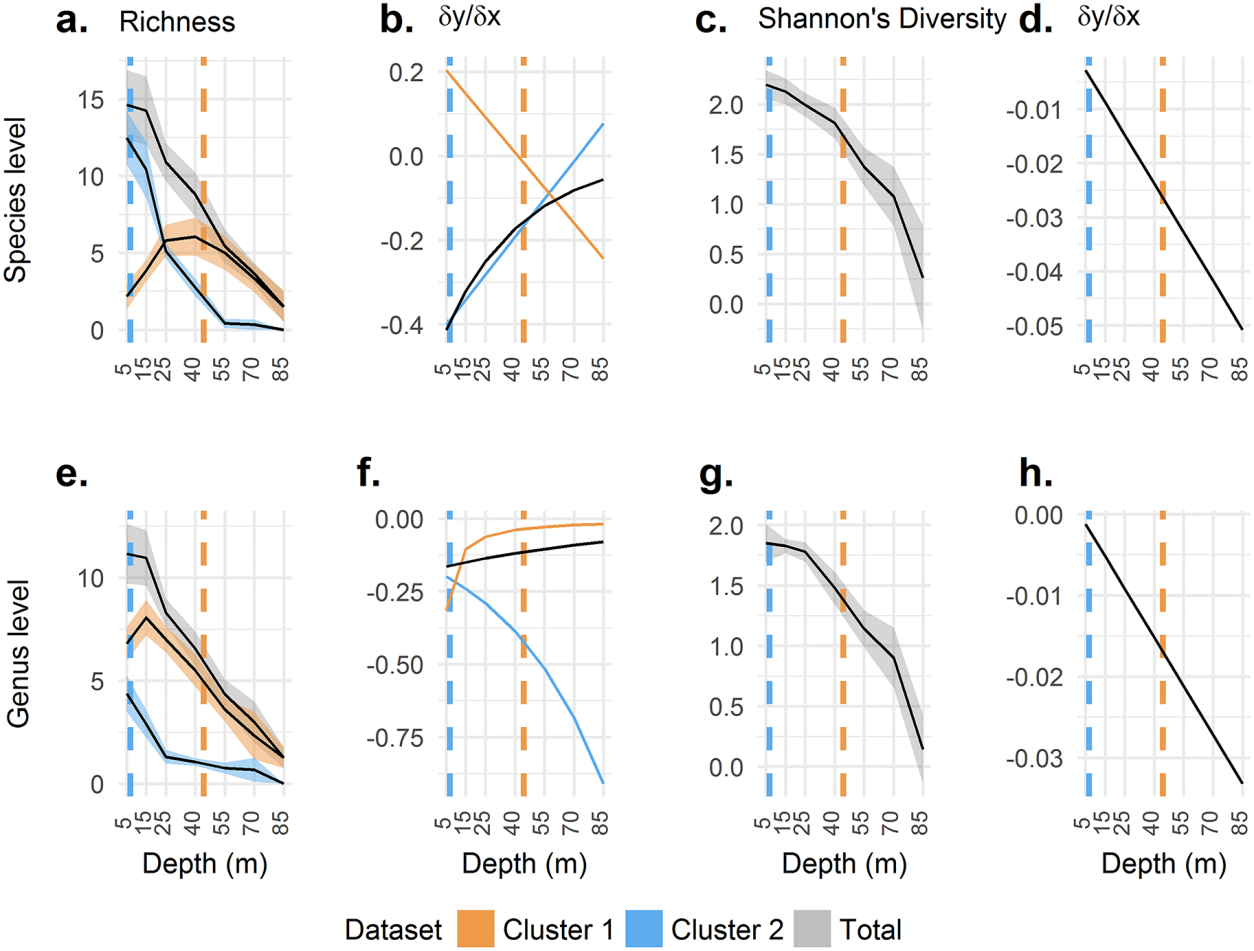
Biodiversity metrics and their relationships with depth. Comparison of different metrics for detecting changes in reef structure. Data are pooled from all sites. Shaded areas are one standard error from the mean (the denominator of the equation was the number of sites sampled at a given depth). The colour coding of the shading represents assemblages consistent with previous plots. Dashed lines are the dataset average boundaries for the deepest transects statistically significantly aligning as shallow and the shallowest transects similarly aligning as deep. Other biodiversity measures and rates of change are plotted for comparison. Note other metrics show no obvious features to demark a switch in community composition.

Fig 6 compares the trends detected using different metrics at differing levels of taxonomic resolution. Fig 6a presents scleractinian species richness within assemblages plotted with depth. Total species richness of scleractinians declined steadily with depth yet the pattern does not hold true for each assemblage. The shallow assemblage showed a rapid decline with depth while the ‘deep’ assemblage exhibits a low humped relationship. Shannon’s diversity drops at an increasing rate with depth, with no obvious feature coinciding with changes in community detected by the ordination approach.

An exponential trend line for the total richness data was appropriate with an *R*^2^ of 0.6575 at species level and 0.7408 at genus level. The differentials were dy/dx = -0.46823e-^0.025x^ for species and dy/dx = -0.17172e-^0.009x^ for genus. The estimated rate of change in richness revealed a faster loss of taxa in the shallows than at depth; this relationship was more marked for species than genera.

For comparison, rates of change in the assemblages defined by K-means clustering were calculated from trend lines (Y = -0.0028X^2^ + 0.2323X + 1.1877 and Y = 9.5235e-^0.019X^), with *R*^*2*^ of 0.354 and 0.5659 for cluster one at species and genus level respectively. Differentials were dy/dx = -0.0056X +0.2323 and dy/dx = -0.1809465e-^0.019X^. For cluster two as above, the trend lines (Y = 0.003X^2^–0.4323X + 14.998 and Y = -1.576ln(X) + 6.8803) had *R*^*2*^ of 0.7405 and 0.5763 yielding dy/dx = 0.006X -0.4323 and dy/dx = -1.576/X at species and genus level.

A second order polynomial was fitted to the Shannon’s diversity data with an *R*^2^ of 0.67 at species level and 0.7152 at genus level, dy/dx = -0.0006X + 0.0002 for species and dy/dx = -0.0004X + 0.0008 for genus. The rate of change in Shannon’s biodiversity became increasingly negative with depth. Interestingly the patterns in all metrics represented in Fig 6 are largely preserved when taxonomic resolution is reduced to the level of the genus, despite the difficulties of mapping Fig 4b. There is no evidence in Fig 6 for peaks in richness or biodiversity, of the total community, which may be expected under an intermediate disturbance hypothesis [49] or indicative of zones of faunal transition. Similarly rates of change fail to equal 0 or peak at any boundary other than for species cluster 1 with the mesophotic boundary. This relationship, however, is based on the worst fitting model of change and requires the ordination approach to identify the cluster.

## Discussion

The analysis presented here generates a representation of vertical changes in scleractinian community assemblage, only informed by patterns of co-occurrence and abundance, yet able to recognise distinct MCE and shallow communities regardless of depth. For the species-level PCoA the depths of occurrence for the two assemblages and transition region loosely coincide with currently held boundaries [4]. However, on transects for Rocky Point and Raggedy Cay, communities identified as shallow or MCE were on one occasion each separated by 10m of water vertically, demonstrating remarkable changes in community structure with small changes in depth (Fig 5). For other patches of reef, the same difference in community was only detected over 50m vertically at Rocky Point and The Maze (Fig 4a). This approach allows the reef community to inform on the type of reef viewed, as opposed to following a set depth limit. These observations highlight the importance of formalising a mobile definition for MCEs capable of incorporating unusual reefs [19].

The changes in scleractinian community structure indicate a loss of shallow specialists and rising dominance of depth-generalists with increasing depth. We see no evidence here for a MCE specialist assemblage based on a definition of typically occurring below 30m, as we record only one species range beginning deeper than this limit. Depth-generalists may be excluded by competitive influences in the shallows, either mediated by photosynthesis and sensitivity to light [50], or resistance to water movement [51]. This is supported by the depth ranges of species assigned to each assemblage (Fig 5), the lack of new species in the species list of our study to 85m in comparison to a study surveying to only 18m a few years prior [52] and the differing relationships between the two identified assemblages with respect to species richness and depth (Fig 6a), as well as more general patterns across taxa studied elsewhere [13]. Unfortunately, this study is unable to provide the environmental data required to fully explain the patterns in community observed. Our study recorded 41 species of scleractinians in comparison to 46 in an earlier study to a maximum depth of 18 m [52]. Scaps & Saunders reported two *Scolymia* species; in this study *Scolymia* was identified to genus level. All other species present in the earlier study but not reported here were classified as ‘uncommon’ or ‘rare’. It is possible the lack of these uncommon species is a result of differing methodology. Scaps & Saunders employed roving diver surveys to identify as many species as possible, while we used transects to standardise survey areas for quantitative analysis.

Patterns in benthic coverage with depth were variable between sites, likely affected by site-specific histories of anthropogenic impact, prevailing environmental conditions, and topography. Though there is no data to support these expectations at Utila, such factors may mask generalised patterns associated with increasing depth [6,30]. Sponge cover remained largely constant with depth at all sites except Raggedy Cay where it increased with depth (Fig 2). All sites share an increasingly fragmented reef structure with increasing depth, represented by increasing sand cover (Fig 1). Despite the potential for systematic over-estimates of scleractinian cover, the patterns across the depth gradient presented here agrees with an earlier transect-based survey in 2014 to a maximum depth of 40m [6], though our estimates of scleractinian cover are substantially higher. This was likely caused by preferential analysis of scleractinians if they appeared adjacent to the transect. Though mature scleractinian reef structure ended at 55m for Utila, and scleractinians were not seen below 85m (all colonies 70 – 85m were small encrusting patches), other Caribbean reefs have dense *Agaricia* beds at 85m [53] and Scleractinia recored as deep as 107m [54]. Our observations while conducting surveys around Utila suggest some MCEs may have suffered high scleractinian mortality at their deepest extents. Mature agariciid reefs may have once existed to at least 70m deep and have since become increasingly shallow (S4 Fig).

We need to improve our understanding of mesophotic species to accurately predict future reef change. The shallow bias of coral biology [55] may have missed a major feature of community structure in coral reefs. Our results suggest, for Utila, Scleractinia should be broadly classed as shallow specialists and depth generalists. This pattern was recently revealed to apply more generally across taxa as well [13]. Past physiological data also hints at a further subdivision within the depth-generalists of those utilising light energy (phyto-generalists) such as *Montastraea cavernosa*, able to promiscuously switch symbionts [7,56], and species such as *Agaricia lamarcki* (trophic-generalists) possibly able to rely on heterotrophic subsidy to offset light levels [57]. Teasing apart reef community structure and future change will require an understanding of the differing responses of these three groups of coral; shallow, phyto-generalists, and trophic-generalists, to a variety of pressures, and the competitive influences between them. Coral reefs may not be committed to a gloomy future if it is possible that the relative abundance of these three groups of coral will shuffle. To assess this, and move towards explaining the ecological structure of coral reefs across their whole vertical range, more physiological work is required. Determining how a broad suite of taxa satisfy their calorific demands, their relationship with zooxanthellae, growth rates and competitive abilities will lead to explanations of ecology rather than documentation of patterns. This could not only improve our understanding of coral reefs from an ecological view, but also their conservation. Understanding the drivers of coral distribution may help improve the choices of species targeted for nursery schemes by matching physiology to future conditions. It may also help us in identifying which species are particularly in need of future protection because of limited plasticity.

From the perspective of the DRRH, our results raise two questions of interest. Firstly, how quickly does the decline in shallow assemblage richness occur with depth at a location? Answering this will reveal whether depth can afford protection for shallow species [13]. When comparing between sites, sites where the decline in shallow assemblage richness and the potential competitive influences with depth occurs more slowly are more likely to satisfy the requirements of the DRRH. Secondly, the abundance of the depth-generalist assemblage peaks after the rapid decline of the shallow assemblage, potentially implying competitive interactions. Understanding whether the generalist species can survive in the shallows in the absence of competition with shallow specialists will reveal whether the DRRH could work, but may result in a phase shift on the reef from one scleractinian assemblage to another.

We chose to focus on scleractinians when considering possible definitions of MCEs for several reasons, though we accept patterns in other taxa may vary and are of interest. The advantages with benthic organisms such as scleractinians include that they are static, attract other associated fauna, and they may offer indications of change under varying environments. There is a lag time between changes in environmental conditions and changing reef structure dictated by rates of recruitment, growth and death of corals in now unfavourable habitats. Rather than a disadvantage we believe this offers opportunity for the detection of natural experiments [24]. When the environment changes, the time window before the community shifts in response provides an opportunity to monitor the effects of unfavourable conditions on these reefs, revealing potential future trends. These opportunities may be missed when definitions are based on mobile taxa.

Despite this, a major challenge of a definition based on scleractinian distribution is the required analysis of species-level community structure. Not every researcher interested in MCEs necessarily has the time or knowledge to conduct a similar study simply to detect which patches of reef are MCEs. Unfortunately, as demonstrated here (Fig 4), reducing the level of taxonomic accuracy used only reduces the ability of the analysis to statistically detect patterns in reef community structure. This is therefore not an option for making the analysis tractable for non-coral researchers. We attempted to select an indicator taxon, the family Agariciidae, to reduce the number of species a non-specialist would need to be able to identify. This failed to resolve any transects as belonging to a distinct assemblage as the three clusters resolved overlap in their distribution. The member species of this family are also similar in their morphology adding difficulty for the non-specialist.

Before wider use, this method would need to be applied more broadly through the Caribbean for validation, and the different pool of species present in the Indo-Pacific poses a problem for direct comparison. It may be that patterns in community structure remain the same in the Indo-Pacific despite taxonomic composition changes between the two regions because of biogeography. However, the degree of taxonomic resolution possible in field studies in the Indo-Pacific is generally lower than in Caribbean studies, possibly limiting the power of analysis in the former. We would encourage other researchers to apply this method to their own data to see if the patterns hold true. Other methods for detecting vertical reef structure also have flaws (Table 1) and utility must be balanced with accuracy. To this end, Fig 6 shows how other biodiversity metrics capture the patterns of the ordination analysis. Reducing taxonomic resolution was explored to improve the accessibility of a new definition. Analysis at the level of the genus may be subject to lower error and requires a less detailed knowledge of the Scleractinia. In the Indo-Pacific with greater diversity in scleractinian genera, genus-level identification may be sufficient to detect patterns.

Richness and rates of change as opposed to biodiversity and ordination techniques were explored as these do not require abundance data. This allows quicker sampling protocols such as roving diver surveys rather than quantitative transects. Species richness has been widely used as it is less labour intensive and does not require a strictly quantitative sampling methodology provided effort is standardised. We show here, however, that it is possible to miss structure if species identities, and so community turnover, are not taken into account (Fig 6). Biodiversity indices may similarly fail to detect changes in community structure while requiring the same data to be collected as for the ordination-based approach. Rates of change in richness and Shannon’s diversity failed to show any obvious features coinciding with community boundaries detected by ordination approaches. Interestingly, however, these approaches revealed a similar relationship with depth when taxonomic resolution was reduced. It may be possible to recreate the groupings returned here by K-means clustering by identifying two groups of scleractinia with differing depth ranges using Fig 5 with the statistical analysis of depth ranges for support. This would allow for a method approximating the ordination without the need for abundance data.

## Conclusions

It is clear that traditional biodiversity measures and absolute depth limits are insufficient for defining vertical biological community zonation across shallow to mesophotic reef gradients. We therefore present a methodology for detecting this zonation and reveal changes in the rate of transition with depth between reefs located geographically within 200m of each other. We accept further discussion is needed to improve on the amount of data required to accurately define these ecosystems for those without taxonomic expertise. It may be possible to generate workable definitions without the need for abundance data if rapid assessment is required; however, doing so removes the ability to detect transitional zones. Despite this, if the main questions of interest are ‘how deep do shallow specialists descend?’ to answer questions regarding the DRRH, or ‘where do MCEs start’ for zone-specific work, this can be achieved by ranking species by depth range to identify the lower depth boundary of the shallow cluster. Other researchers should also investigate other potential definitions to inform debate.

We found evidence of formerly extensive MCEs at depths of 70m on the north shore of Utila, and that reefs with similar species compositions are now found at approximately 55m depth. These MCEs appear to be populated by depth generalists which may rely either on heterotrophic subsidy or the manipulation of photosynthetic adaptations to accommodate their depth range. The transition between reef types is characterised by the loss of shallow-specialists and subsequent increase depth-generalist dominance. Further work on the physiology of Scleractinia on MCEs will help to determine the mechanisms behind such ecological patterns.

## Supporting information

S1 Table Study scleractinia species list. All Identified Scleractinia around the island of Utila from 5 m to 85 m depth during the survey period. The taxonomic hierarchy here defines the species, genus and family level analyses conducted as part of the study. *Millepora* was included as a group in all analyses as a common hermatype, *Scolymia* spp. was included as a single taxon in the species level analysis. The total number of species recorded was 41 across 22 genera and 8 families.
(PDF)

S2 Table Locations significantly associated with scleractinian assemblages. Transect IDs are listed with the P values returned from Defrene-Lengendre indicator species analysis performed on three levels of taxonomic resolution. Only P values <0.05 are reported. Underlined numbers are the cluster identity the transect aligns with.
(PDF)

S1 Fig Shepard plot for Fig 3. Showing the distance between points in the ordination of Fig 3 and the correlation with observed dissimilarity between points. The ordination appears to faithfully reflect the dissimilarity between transects with a non-metric fit R2 = 0.976.
(TIF)

S2 Fig Scleractinia assemblage detection. The left pane shows a K means clustered Principal Co-ordinate analysis. Polygons enclose points within a cluster. The right pane shows the Calinski criterion for a different proposed number of clusters to be fitted to the data. The largest value was selected as the best choice of number of clusters, denoted by a hollow point. (a.) Analysis based on species level ID. (b.) Analysis based on genus level ID. (c.) Analysis based on family level ID.
(TIF)

S3 Fig Dataset average depth ranges of scleractinia genera. Box plots of genera depth ranges with data pooled across sites. Box plots are coloured internally to reveal the assemblage they belong to. All box plots on the darker background belong to Cluster 1, the lighter background denotes cluster 2. Lines extend 1.5x the interquartile range or to the last observation. Points are outliers beyond this limit.
(TIF)

S4 Fig Evidence of past mature *Agaricia* colonies at 70 m depth at TMA. Live and inferred dead portions are highlighted. Old colony extent is evident from preserved corallite patterns in the substrate. The bare skeleton has not been heavily fouled.
(TIF)

S1 CSV Matrix of scleractinia counts by species. The data used for the ordination analyses. Count data of Scleractinia species identified during the sampling period at 5 different dive sites around the Island of Utila, Honduras. Transects were collected from 5 to 85m deep with 4 replicates per location.
(CSV)

S2 CSV Percentage cover estimates for benthic categories. Raw data as displayed in Fig 2.
(CSV)

S1 code Generalised R code for K-means clustering and generating Fig 4. This R code will take an ecological matrix as detailed in the header, perform K-means clustering and extract the optimal solution. Associations of species are plotted and Defrene-Legendre analysis indicates how similar the species on a given transect are to identified assemblages. Finally this is visualised as a ‘map’ of clusters as in Fig 4. We hope this will act as a start point for others to conduct similar searches for a mobile depth limit for mesophotic reefs.
(R) Click here for additional data file.
