# Supplementary material for: Using light-dependent scleractinia to define the upper boundary of mesophotic coral ecosystems on the reefs of Utila, Honduras
Source: PLoS One. 2017 Aug 15;12(8):e0183075. doi: 10.1371/journal.pone.0183075 (PMC5557359; doi:10.1371/journal.pone.0183075)
Supplement: S1 Table — All Identified Scleractinia around the island of Utila from 5 m to 85 m depth during the survey period. The taxonomic hierarchy here defines the species, genus and family level analyses conducted as part of the study. Millepora was included as a group in all analyses as a common hermatype, Scolymia spp. was included as a single taxon in the species level analysis. The total number of species recorded was 41 across 22 genera and 8 families. (PDF) [file pone.0183075.s001.pdf]

S1 Table. *Study Scleractinia species list*

All Identified Scleractinia around the island of Utila from 5 m to 85 m depth during the survey period. The taxonomic hierarchy here defines the species, genus and family level analyses conducted as part of the study. Millepora was included as a group in all analyses as a common hermatype, Scolymia spp. was included as a single taxon in the species level analysis. The total number of species recorded was 41 across 22 genera and 8 families.

Family	Genus	Species	Family	Genus	Species
Acroporidae	Acropora	cervicornis	Faviidae	Montastraea	cavernosa
		palmata		Orbicella	annularis
Agariciidae	Agaricia	fragilis			faveolata
		grahamae			franksi
		lamarcki	Meandrinidae	Dendrogyra	cylindrus
		undata		Dichocoenia	stokesii
	Helioseris	cucullata		Eusmilia	fastigiata
	Undaria	agaricites		Meandrina	meandrites
		humilis	Mussidae	Scolymia	Spp.
		Tenuifolia		Isophyllia	rigida
Astrocoeniidae	Madracis	auretenra		Mycetophyllia	aliciae
		decactis			ferox
		Formosa			lamarckiana
		pharensis			reesi
		senaria	Poritidae	Porites	astreoides
	Stephanocoenia	intersepta			furcata
Faviidae	Colpophyllia	natans			porites
	Diploria	labyrinthiformis	Siderastreidae	Siderastrea	radians
	Favia	fragum			siderea
	Manicina	areolata			
