# Supplementary material for: Using light-dependent scleractinia to define the upper boundary of mesophotic coral ecosystems on the reefs of Utila, Honduras
Source: PLoS One. 2017 Aug 15;12(8):e0183075. doi: 10.1371/journal.pone.0183075 (PMC5557359; doi:10.1371/journal.pone.0183075)
Supplement: S2 Table — Transect IDs are listed with the P values returned from Defrene-Lengendre indicator species analysis performed on three levels of taxonomic resolution. Only P values <0.05 are reported. Underlined numbers are the cluster identity the transect aligns with. (PDF) [file pone.0183075.s002.pdf]

S2 Table. Defrene-Legendre indicator analysis results

Transect IDs are listed with the P values returned from Defrene-Lengendre indicator species analysis performed on three levels of taxonomic resolution. Only P values <0.05 are reported. Underlined numbers are the best-fitting cluster identity the transect indicates.

Transect	Species	Genus	Family	Transect	Species	Genus	Family
CV.5m.1		2 0.005		RC.55m.1	1 0.006		4 0.047
CV.5m.2	2 0.025			RC.55m.2	1 0.005	2 0.044	
CV.5m.3	2 0.032			RC.55m.3	1 0.005		
CV.5m.4	2 0.002			RC.55m.4	1 0.004	2 0.031	
CV.15m.2		2 0.029		RP.5m.1	2 0.001		
CV.15m.3		2 0.015		RP.5m.2	2 0.001		
CV.15m.4		2 0.001		RP.5m.4	2 0.003		
CV.25m.1		2 0.007		RP.15m.1	2 0.003	2 0.008	
CV.25m.2		2 0.007		RP.15m.2	2 0.002	2 0.012	
CV.25m.3		2 0.001		RP.15m.3	2 0.014	2 0.003	
CV.25m.4		2 0.002		RP.15m.4	2 0.007	2 0.034	
CV.40m.1	1 0.047	2 0.005		RP.25m.1		2 0.001	
CV.40m.2	1 0.023	2 0.013		RP.25m.2		2 0.001	
CV.40m.3		2 0.027		RP.25m.3		2 0.006	
CV.40m.4	1 0.021	2 0.032		RP.25m.4		2 0.001	
LB.5m.1	2 0.018			RP.40m.1		2 0.006	
LB.5m.3	2 0.014			RP.40m.2		2 0.001	3 0.023
LB.5m.4	2 0.016			RP.40m.3	1 0.001	2 0.001	
LB.15m.2		2 0.01		RP.40m.4		2 0.006	
LB.15m.3		2 0.025		RP.55m.1	1 0.001	2 0.003	
LB.15m.4		2 0.003		RP.55m.2	1 0.016		
LB.25m.1		2 0.005		RP.55m.3			3 0.05

S2 Table continued

Transect	Species	Genus	Family	Transect	Species	Genus	Family
LB.25m.2		2 0.002		TMA.5m.1	2 0.001		
LB.25m.3		2 0.006		TMA.5m.2	2 0.002		
LB.25m.4		2 0.001	4 0.035	TMA.5m.3	2 0.03		
LB.40m.1	1 0.002	2 0.016	3 0.035	TMA.5m.4	2 0.001		
LB.40m.2	1 0.001	2 0.003		TMA.15m.1		2 0.002	
LB.40m.3	1 0.001	2 0.012		TMA.15m.2	2 0.049	2 0.013	
LB.40m.4	1 0.002	2 0.003		TMA.15m.3		2 0.005	
RC.5m.1		2 0.012		TMA.15m.4		2 0.003	
RC.5m.2	2 0.023			TMA.25m.1		2 0.001	
RC.5m.4	2 0.004	2 0.032		TMA.25m.2		2 0.007	
RC.15m.1		2 0.013		TMA.25m.3		2 0.033	
RC.15m.2	2 0.029		2 0.041	TMA.25m.4		2 0.012	
RC.15m.3		2 0.001		TMA.40m.1	1 0.004	2 0.001	
RC.15m.4		2 0.004		TMA.40m.3		2 0.042	
RC.25m.2		2 0.001		TMA.40m.4		2 0.033	
RC.25m.3	1 0.015	2 0.001		TMA.55m.1	1 0.004	2 0.044	
RC.25m.4		2 0.003		TMA.55m.2	1 0.013		
RC.40m.1	1 0.044	2 0.004	4 0.038	TMA.55m.3	1 0.015		
RC.40m.2		2 0.001	4 0.026	TMA.55m.4	1 0.025		
RC.40m.3		2 0.037	4 0.013	TMA.70m.2	1 0.009		
RC.40m.4	1 0.01		4 0.038				
