# Supplementary figures and images for: Using light-dependent scleractinia to define the upper boundary of mesophotic coral ecosystems on the reefs of Utila, Honduras
Source: PLoS One. 2017 Aug 15;12(8):e0183075. doi: 10.1371/journal.pone.0183075 (PMC5557359; doi:10.1371/journal.pone.0183075)

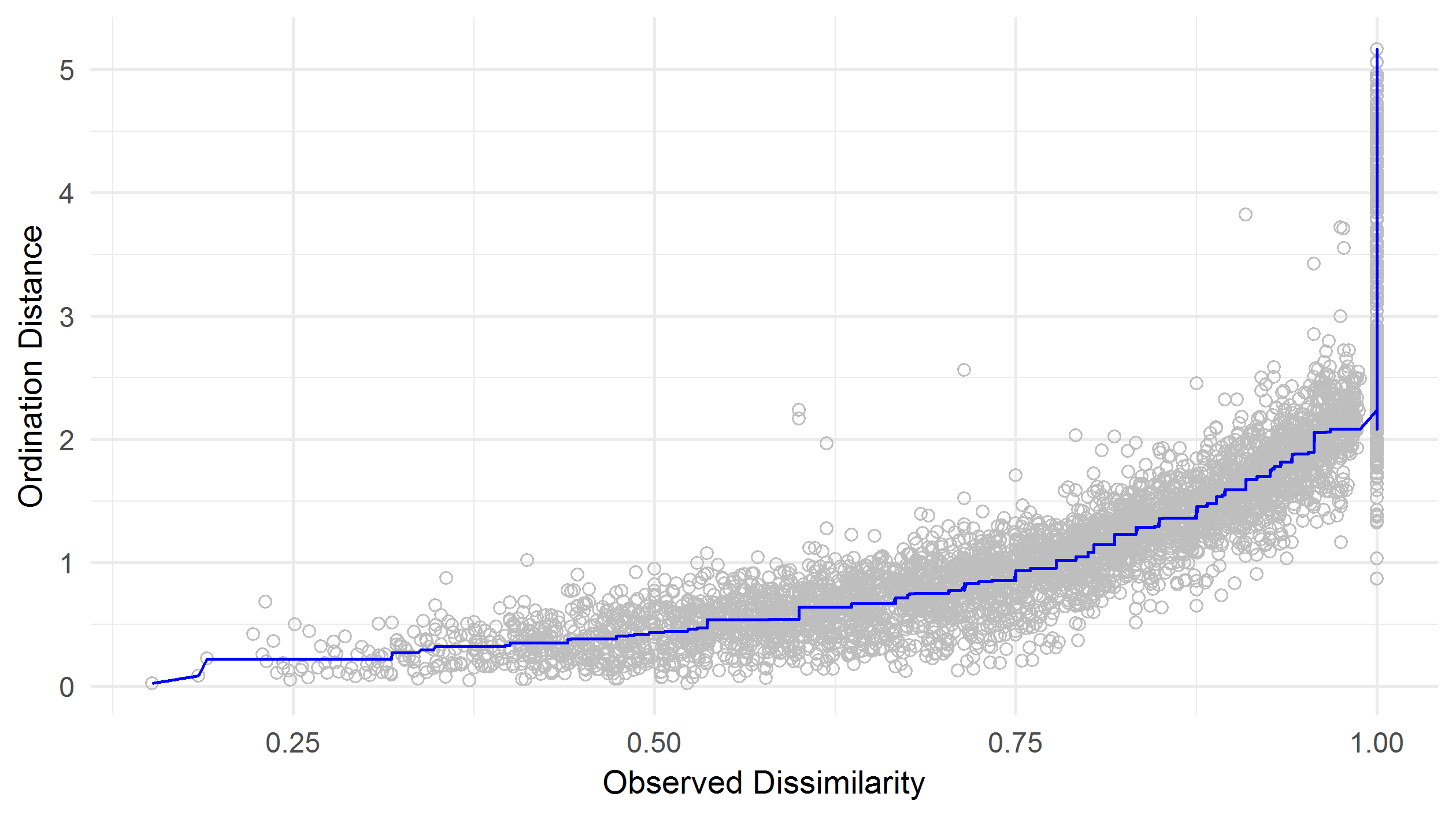

Supplement: S1 Fig — Showing the distance between points in the ordination of Fig 3 and the correlation with observed dissimilarity between points. The ordination appears to faithfully reflect the dissimilarity between transects with a non-metric fit R2 = 0.976. (TIF) [file pone.0183075.s003.tif]

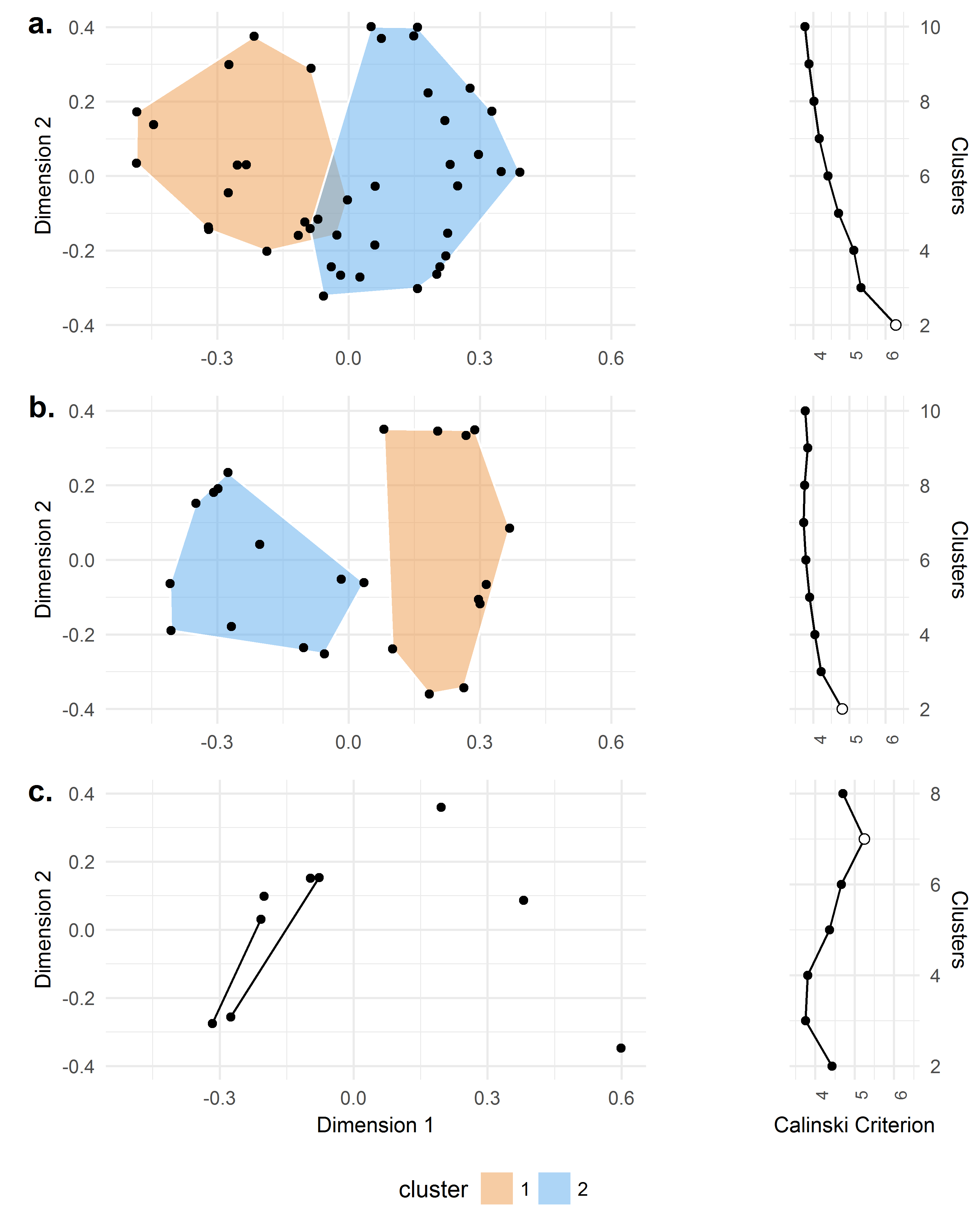

Supplement: S2 Fig — The left pane shows a K means clustered Principal Co-ordinate analysis. Polygons enclose points within a cluster. The right pane shows the Calinski criterion for a different proposed number of clusters to be fitted to the data. The largest value was selected as the best choice of number of clusters, denoted by a hollow point. (a.) Analysis based on species level ID. (b.) Analysis based on genus level ID. (c.) Analysis based on family level ID. (TIF) [file pone.0183075.s004.tif]

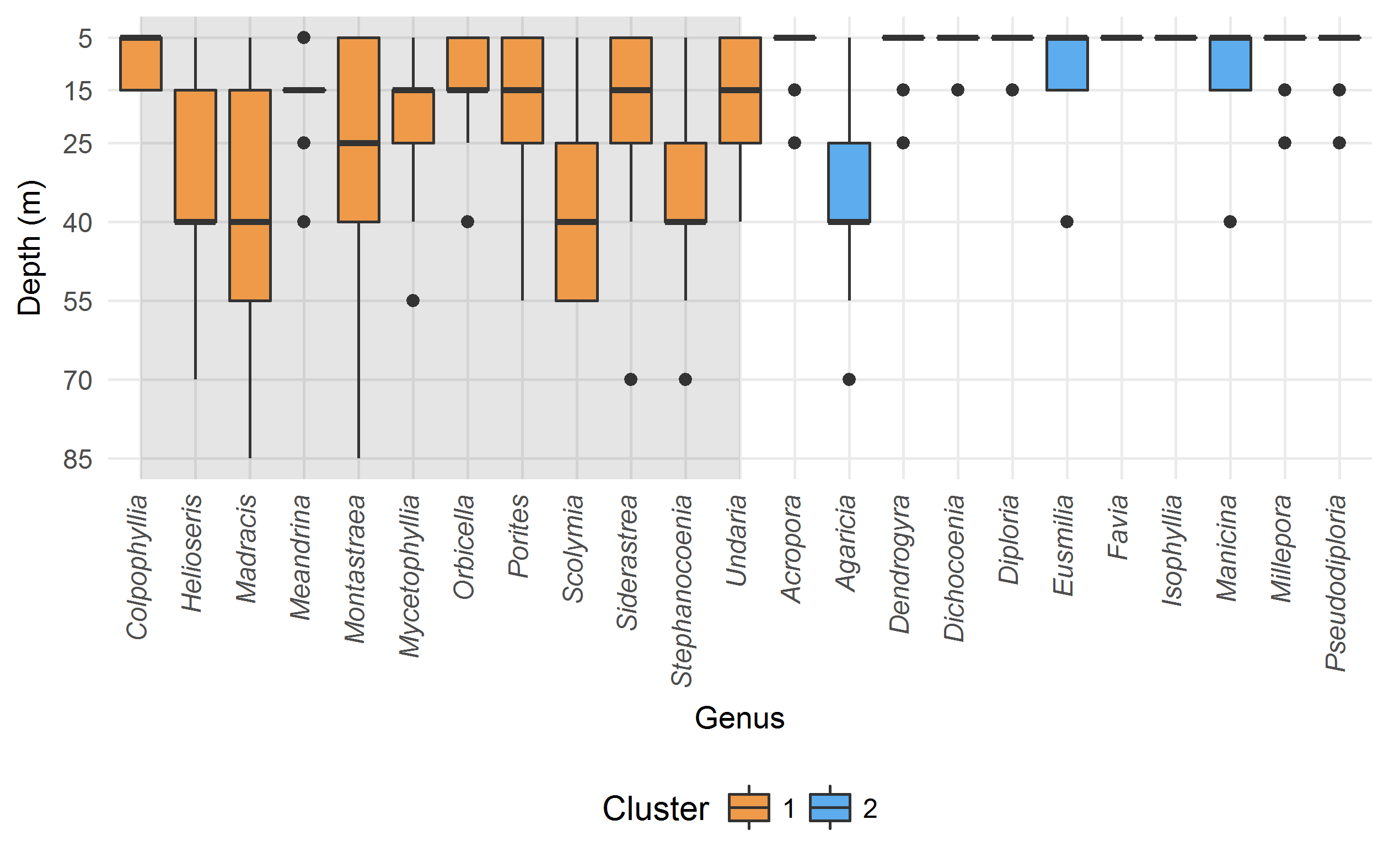

Supplement: S3 Fig — Box plots of genera depth ranges with data pooled across sites. Box plots are coloured internally to reveal the assemblage they belong to. All box plots on the darker background belong to Cluster 1, the lighter background denotes cluster 2. Lines extend 1.5x the interquartile range or to the last observation. Points are outliers beyond this limit. (TIF) [file pone.0183075.s005.tif]

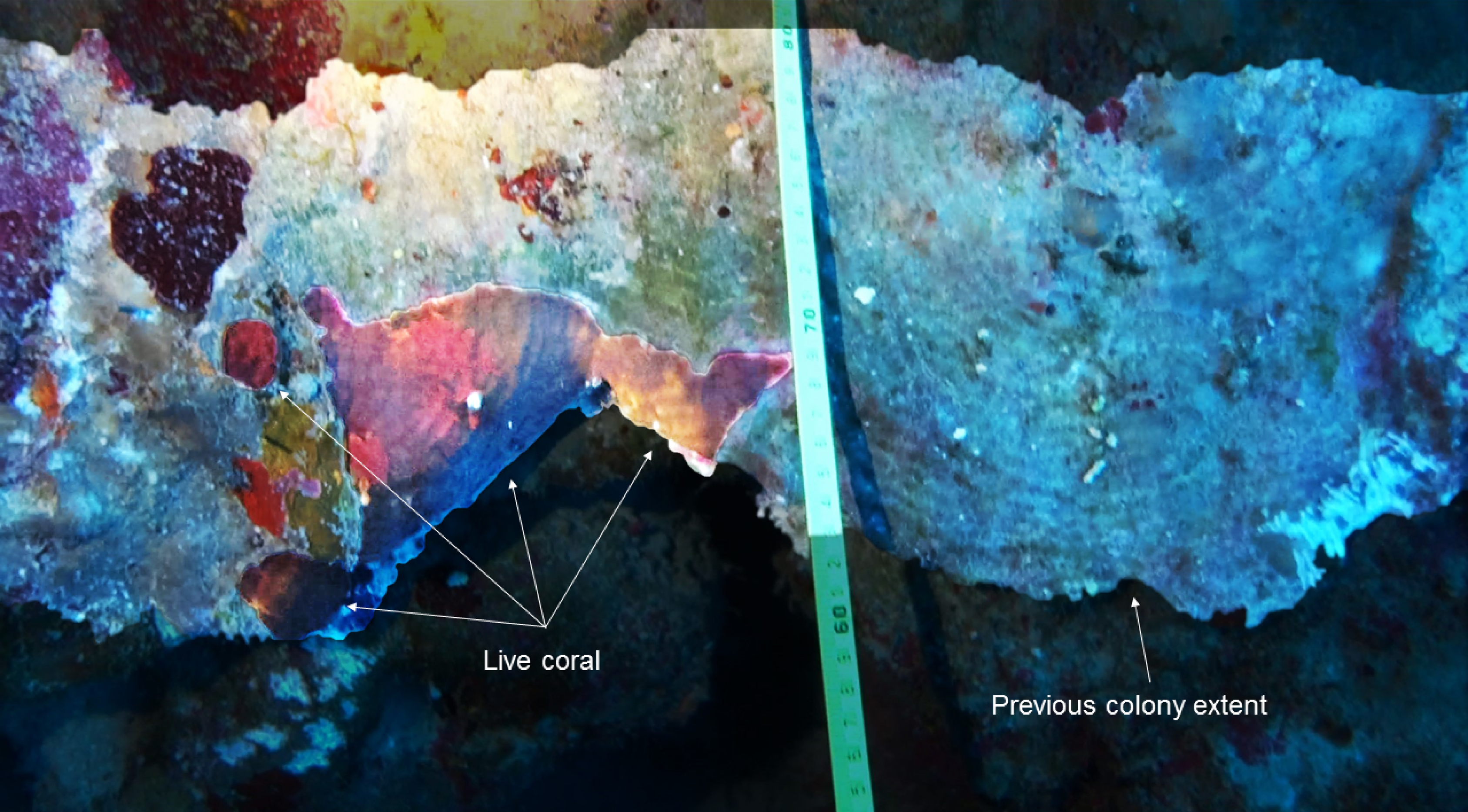

Supplement: S4 Fig — Live and inferred dead portions are highlighted. Old colony extent is evident from preserved corallite patterns in the substrate. The bare skeleton has not been heavily fouled. (TIF) [file pone.0183075.s006.tif]
